# Efficacy of Lignocaine Jelly Versus Water-Based Lubricant in Preventing Postoperative Airway Morbidity After Endotracheal Intubation: A Prospective Comparative Study

**DOI:** 10.7759/cureus.92871

**Published:** 2025-09-21

**Authors:** Srirupa Mandal, Sourabh Roy, Anuradha Mitra, Anjum Naz, Madhumita Bhakta, Abhimanyu Behera

**Affiliations:** 1 Anesthesiology, All India Institute of Medical Sciences, Kalyani, IND; 2 Anesthesiology, KPC Medical College and Hospital, Kolkata, IND; 3 Community Medicine, SLN Medical College and Hospital, Koraput, IND; 4 Community Medicine, MKCG Medical College and Hospital, Berhampur, IND

**Keywords:** 2% lignocaine, endotracheal intubations, intubation complication, lubricant, postoperative sore throat

## Abstract

Background: Postoperative sore throat, cough, and hoarseness of voice are common sequelae of endotracheal intubation. These symptoms, although self-limiting, are distressing to patients and may affect their perioperative experience and satisfaction. Various methods have been explored to reduce these complications, including the lubrication of the endotracheal tube (ETT) with local anesthetics or simple lubricating agents. However, evidence regarding the comparative efficacy of lignocaine jelly and water-based lubricants remains inconclusive.

Aim: To compare the efficacy of lignocaine jelly and water-based lubricating jelly applied over ETTs in reducing postoperative sore throat, cough, and hoarseness of voice in patients undergoing elective surgery under general anesthesia.

Methods: This prospective, comparative study was conducted on 120 adult patients (American Society of Anesthesiologists (ASA) I-II, aged 18-60 years) posted for elective surgeries under general anesthesia with endotracheal intubation. Patients were randomly allocated into two groups: Group L (n=60), where 2% lignocaine jelly was applied to the ETT, and Group W (n=60), where water-based lubricating jelly was applied. Standardized anesthesia techniques were used in both groups. Postoperative assessment of sore throat, cough, and hoarseness of voice was carried out at 1, 6, 12, and 24 hours using a direct questionnaire and graded scoring system. Data were analyzed using SPSS (version 27) (IBM Corp., Armonk, NY), with chi-square and t-tests applied; p<0.05 was considered statistically significant.

Results: Both groups were comparable in demographic characteristics and intraoperative variables. The overall incidence and severity of postoperative sore throat, cough, and hoarseness were lower in the water-based jelly group compared to the lignocaine jelly group, with statistically significant differences noted particularly at early postoperative intervals. By 24 hours, most symptoms had subsided in both groups.

Conclusion: Water-based lubricating jelly applied over the ETT is more effective than lignocaine jelly in reducing the incidence and severity of postoperative sore throat, cough, and hoarseness of voice. Being inexpensive, widely available, and devoid of potential systemic side effects of lignocaine, it may be recommended as a safer alternative for routine clinical practice.

## Introduction

Endotracheal intubation remains an integral component of modern anesthetic practice, ensuring airway patency, preventing aspiration, and facilitating mechanical ventilation during surgical procedures. Despite its undeniable benefits, intubation is not without complications. Among the most frequent and bothersome sequelae in the immediate postoperative period are sore throat, cough, and hoarseness of voice. These symptoms, although self-limiting, are often distressing to patients and may influence postoperative satisfaction, particularly in day-care or short-stay surgical settings where recovery experience plays a pivotal role in perceived quality of care [1,2]. The reported incidence of postoperative sore throat varies widely from 20-70% depending on patient, procedural, and anesthetic factors [3]. Pathophysiologically, these symptoms are primarily attributed to mucosal trauma, ischemia, and inflammatory changes resulting from direct laryngoscopy, passage of the endotracheal tube (ETT), and cuff-induced pressure on the tracheal wall [4]. Additional contributing factors include tube size, cuff design, cuff pressure, duration of intubation, and presence of nasogastric tubes [5]. Given their frequency, these airway morbidities not only prolong discomfort but also complicate recovery in patients undergoing abdominal or thoracic surgery, where effective coughing and phonation are critical [6]. Therefore, strategies aimed at reducing postoperative symptoms, cough, and hoarseness of voice continue to receive attention in anesthesiology research.

Numerous approaches have been evaluated to mitigate airway complications following intubation, including optimization of tube size and cuff pressure, intracuff drug instillation, topical sprays, systemic agents, and lubrication of ETTs with various substances [7]. Among these, lubrication remains a simple, cost-effective, and widely applicable method. Lignocaine jelly, an amide-type local anesthetic, is frequently used to lubricate ETTs due to its mucosal anesthetic properties and potential to attenuate airway reflexes. Studies, however, have yielded conflicting results. Kori et al. reported that lignocaine jelly application increased the severity of sore throat, possibly due to mucosal irritation [8]. Similarly, Soltani et al. observed that lubrication with lignocaine jelly was associated with a higher frequency of cough and sore throat compared to controls [9]. Water-based lubricating gels (e.g., KY jelly), in contrast, are inert, non-irritant, and commonly used to reduce friction during intubation. Some randomized controlled trials have demonstrated that water-based lubricants are equally effective, if not superior, to lignocaine jelly in minimizing airway complications. Naveed et al. reported significantly lower incidence of postoperative sore throat and cough in the water-based jelly group compared with lignocaine and betamethasone gel groups [7]. Similarly, Doukomo et al. found reduced throat symptoms when KY jelly was used compared to lignocaine jelly [10]. Topical corticosteroid gels such as betamethasone have shown superior efficacy in reducing postoperative sore throat compared with both lignocaine and water-based lubricants, likely due to their anti-inflammatory properties [11,12]. However, their higher cost, potential systemic absorption, and limited availability restrict widespread use, especially in resource-constrained settings. A recent meta-analysis highlighted heterogeneity in trial designs, patient populations, and outcome measures, underscoring the need for further high-quality comparative trials [13].

Despite decades of research, no consensus exists regarding the ideal lubricating agent for ETTs. The available studies are limited by small sample sizes, variable methodology, and conflicting outcomes. Many studies have compared lignocaine jelly with corticosteroid gels, but fewer have directly compared lignocaine with simple water-based lubricants [7,10]. Moreover, the reported findings vary across populations, making it difficult to draw universal conclusions. Importantly, some studies have raised safety concerns with lignocaine jelly, suggesting that its use may paradoxically increase airway irritation and the incidence of sore throat [8,9]. In contrast, evidence supporting the efficacy of water-based lubricants is promising but not yet robust enough to influence standard practice guidelines. Therefore, there exists a clear need to re-examine this question in a systematic, adequately powered, randomized controlled design.

The choice of an appropriate lubricating agent has significant implications for patient comfort, recovery experience, and healthcare quality metrics. Lignocaine jelly, though widely used, may not confer additional benefit and could potentially worsen airway complications. On the other hand, water-based lubricating jelly is inexpensive, inert, widely available, and devoid of systemic side effects. In resource-limited healthcare systems, especially in developing countries, cost-effectiveness and safety of interventions are critical. Evaluating whether a simple water-based lubricant is as effective, or superior, to lignocaine jelly has direct relevance to clinical practice. A well-designed comparative study in an Indian population can provide much-needed clarity on this issue and potentially inform institutional protocols. The present study was designed to compare the efficacy of lignocaine jelly and water-based lubricating jelly in reducing postoperative sore throat, cough, and hoarseness of voice in patients undergoing surgery under general anesthesia with endotracheal intubation and to assess the incidence and severity of these airway complications at specific postoperative intervals (1, 6, 12, and 24 hours), thereby identifying temporal patterns of symptom resolution. Through this study, we aim to generate robust clinical evidence to guide anesthesiologists in selecting the most effective, safe, and practical agent for ETT lubrication, thereby enhancing patient comfort and satisfaction in the postoperative period.

## Materials and methods

This study was designed as a prospective, comparative, double-blind study conducted in the Department of Anesthesiology, KPC Medical College and Hospital, Jadavpur, Kolkata. The work was carried out in the new operating theatre complex, which caters to surgical and orthopedic patients undergoing elective procedures under general anesthesia. The study was conducted over an 11-month period, from March 2019 to January 2020. The study population comprised adult patients aged between 18 and 60 years, belonging to the American Society of Anesthesiologists (ASA) physical status I and II, and posted for elective surgery with an expected duration of less than 240 minutes. The minimum sample size was estimated as 120 subjects to allow comparison of outcomes between the two groups with adequate statistical power. Patients were divided into two groups of 60 each, based on the agent used for ETT lubrication. Group L included patients where the distal 15 cm of the ETT was lubricated with 2% lignocaine jelly, whereas in Group W the tube was lubricated with water-based jelly (KY/Lubic jelly). Patients who met the inclusion criteria were enrolled after pre-anesthetic evaluation and obtaining written informed consent. Those with oral or pharyngeal surgeries, more than two intubation attempts, use of nasogastric tubes or throat packs, active upper respiratory tract infections, or those on chronic steroid therapy were excluded from the study.

All patients received uniform premedication with oral alprazolam (0.5 mg) and ranitidine (150 mg) administered 10 hours before surgery. Anesthetic induction was carried out using fentanyl 2 µg/kg, propofol 1.5-2.5 mg/kg, and succinylcholine 1.5 mg/kg, and intubation was performed with a sterile PVC ETT with a high-volume, low-pressure cuff. An assistant, not involved in intubation, applied the allocated jelly to ensure blinding, while the intubating resident remained unaware of the type of jelly used. Anesthesia was maintained with oxygen, nitrous oxide, sevoflurane, and intermittent doses of atracurium. At the end of surgery, neuromuscular blockade was antagonized with glycopyrrolate and neostigmine, and extubation was performed once the patient was fully awake. Postoperative airway symptoms were assessed using a structured direct questionnaire, adapted from Harding and McVey’s scoring system, which graded sore throat, cough, and hoarseness on a scale of 0-3 [14]. Assessments were made at 1, 6, 12, and 24 hours after extubation by an independent observer who was not involved in patient intubation. Alongside quantitative scores, patient-reported experiences of discomfort were noted to provide qualitative insights into the burden of symptoms, thus adding a mixed-method dimension to the study.

All data were recorded in a predesigned proforma, including demographic characteristics, intraoperative details, and postoperative symptom scores, in MS Excel. Quantitative data were summarized as mean and standard deviation for continuous variables and as counts and percentages for categorical variables using JAMOVI version 2.6.44 (https://www.jamovi.org). Comparisons between groups were made using independent t-tests for continuous variables and Chi-square tests for categorical variables, with a p-value <0.05 considered statistically significant. To ensure reliability of results, intubations were performed by trained residents under supervision following a standardized anesthetic protocol. Data entry was cross-verified by a second investigator to minimize errors. Ethical approval for the study was obtained from the Institutional Ethics Committee of KPC Medical College and Hospital, and informed consent was obtained from each participant. Confidentiality and the right to withdraw without prejudice were respected throughout, in accordance with the Declaration of Helsinki.

## Results

Table 1 presents the comparison of demographic characteristics between the two study groups. The mean age of patients in Group L (lignocaine jelly) was 40.28±8.10 years, while in Group W (water-based jelly) it was 39.02±8.48 years. The difference was not statistically significant (p=0.4042), indicating that the two groups were comparable in terms of age distribution. The gender distribution was also similar across both groups. Group L included 30 males (50%) and 30 females (50%), whereas Group W comprised 34 males (56.7%) and 26 females (43.3%). The difference in gender ratio was not significant (p=0.4642). In terms of physical parameters, the mean height of patients in Group L was 162.47±9.73 cm compared to 161.23±9.03 cm in Group W. Likewise, the mean weight in Group L was 60.23±7.40 kg, whereas in Group W it was 58.97±10.90 kg. Both height and weight comparisons showed no statistically significant differences, with p-values of 0.4700 and 0.4578, respectively. These results confirm that the two groups were well matched with respect to demographic and baseline characteristics. This comparability ensures that any observed differences in postoperative outcomes between the groups can be attributed to the intervention (type of jelly used for ETT lubrication) rather than to underlying demographic variability.

**Table 1:** Comparison of demographic parameters of the patients of the two groups.

Demographic parameters	Group L (n=60)	Group W (n=60)	P-value
Mean age (in years)	40.2833±8.0950	39.0167±8.4763	P=0.4042
Gender (male:female)	30 (50.0%):30 (50.0%)	34 (56.7%):26 (43.3%)	p=0.4642
Height (in cm)	162.4667±9.7258	161.2250±9.0289	P=0.4700
Weight (in kg)	60.2333±7.3953	58.9667±10.9001	P=0.4578

Table 2 shows the postoperative incidence of sore throat, cough, and hoarseness of voice assessed at 1, 6, 12, and 24 hours following extubation in both study groups. At the 1-hour interval, symptoms were most frequent in both groups, with a notably higher incidence in the lignocaine jelly group compared to the water-based jelly group. Specifically, sore throat was reported in 35% of patients in Group L versus 20% in Group W, cough occurred in 28% versus 18%, and hoarseness in 26% versus 20%, respectively. At 6 hours, the overall incidence of symptoms declined in both groups but remained consistently higher in Group L. Sore throat was seen in 30% of patients in Group L compared to 18% in Group W, while cough occurred in 25% and 15% of patients, respectively. Hoarseness followed a similar pattern, with 22% in Group L and 14% in Group W. By 12 hours post-extubation, symptom prevalence further reduced across both groups. Sore throat persisted in 20% of patients in Group L and 10% in Group W, while cough was reported in 15% and 10% of patients, respectively. Hoarseness was noted in 15% of patients in Group L compared to 10% in Group W. At 24 hours, most symptoms had nearly resolved in both groups, though the lignocaine jelly group continued to show slightly higher rates. Sore throat was reported in 10% of patients in Group L compared to 5% in Group W, cough in 8% versus 5%, and hoarseness in 7% versus 5%. Overall, this table demonstrates a consistent trend where the water-based jelly group experienced fewer airway complications at all observed intervals compared to the lignocaine jelly group. The differences were most pronounced in the immediate postoperative period (1-6 hours) and gradually diminished as symptoms resolved by 24 hours. 

**Table 2:** Comparison of postoperative sore throat, cough, and hoarseness of voice at different time intervals between Group L and Group W.

Time (hours)	Sore throat - Group L (%)	Sore throat - Group W (%)	Cough - Group L (%)	Cough - Group W (%)	Hoarseness - Group L (%)	Hoarseness - Group W (%)
1	35	20	28	18	26	20
6	30	18	25	15	22	14
12	20	10	15	10	15	10
24	10	5	8	5	7	5

**Figure 1:**
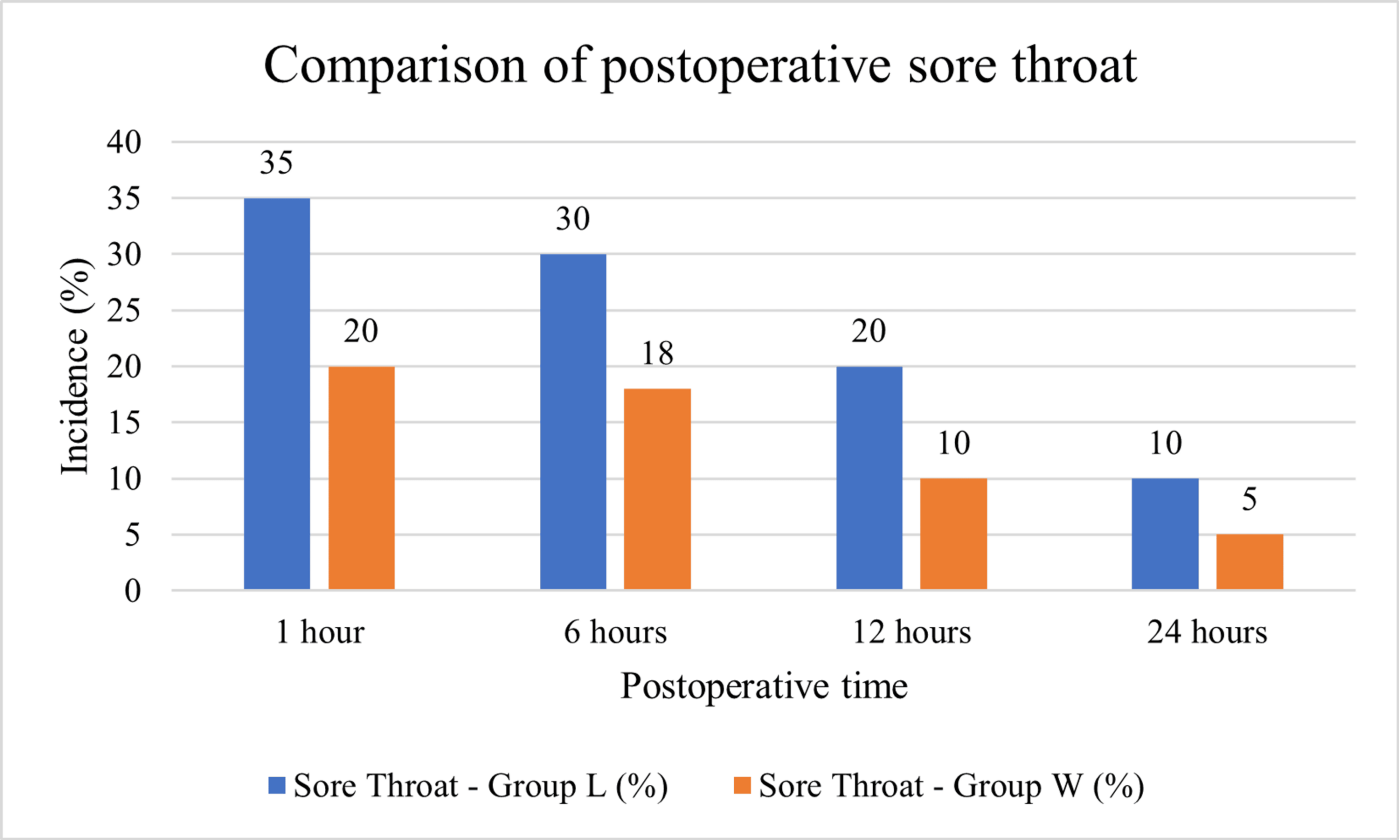
Comparison of the incidence of postoperative sore throat between lignocaine jelly (Group L) and water-based jelly (Group W) at different time intervals.

Figure 1 demonstrates the comparison of postoperative sore throat incidence between the lignocaine jelly group (Group L) and the water-based jelly group (Group W) across different postoperative time intervals. At 1 hour, the incidence of sore throat was highest in both groups, affecting 35% of patients in Group L compared to 20% in Group W. This early postoperative period showed the largest difference between the groups. By 6 hours, the prevalence of sore throat declined to 30% in Group L and 18% in Group W, though the water-based group continued to report fewer symptoms. At 12 hours, the incidence further decreased to 20% in Group L and 10% in Group W, highlighting a consistent trend of lower airway morbidity in patients lubricated with water-based jelly. By 24 hours, the sore throat had almost resolved in both groups, with only 10% of patients in Group L and 5% in Group W still experiencing symptoms. This figure indicates that postoperative sore throat was more frequent and persistent in the lignocaine jelly group, particularly in the immediate hours following extubation. In contrast, water-based jelly was associated with a consistently lower incidence of sore throat across all time points, suggesting its superiority in reducing this common postoperative complication.

**Figure 2:**
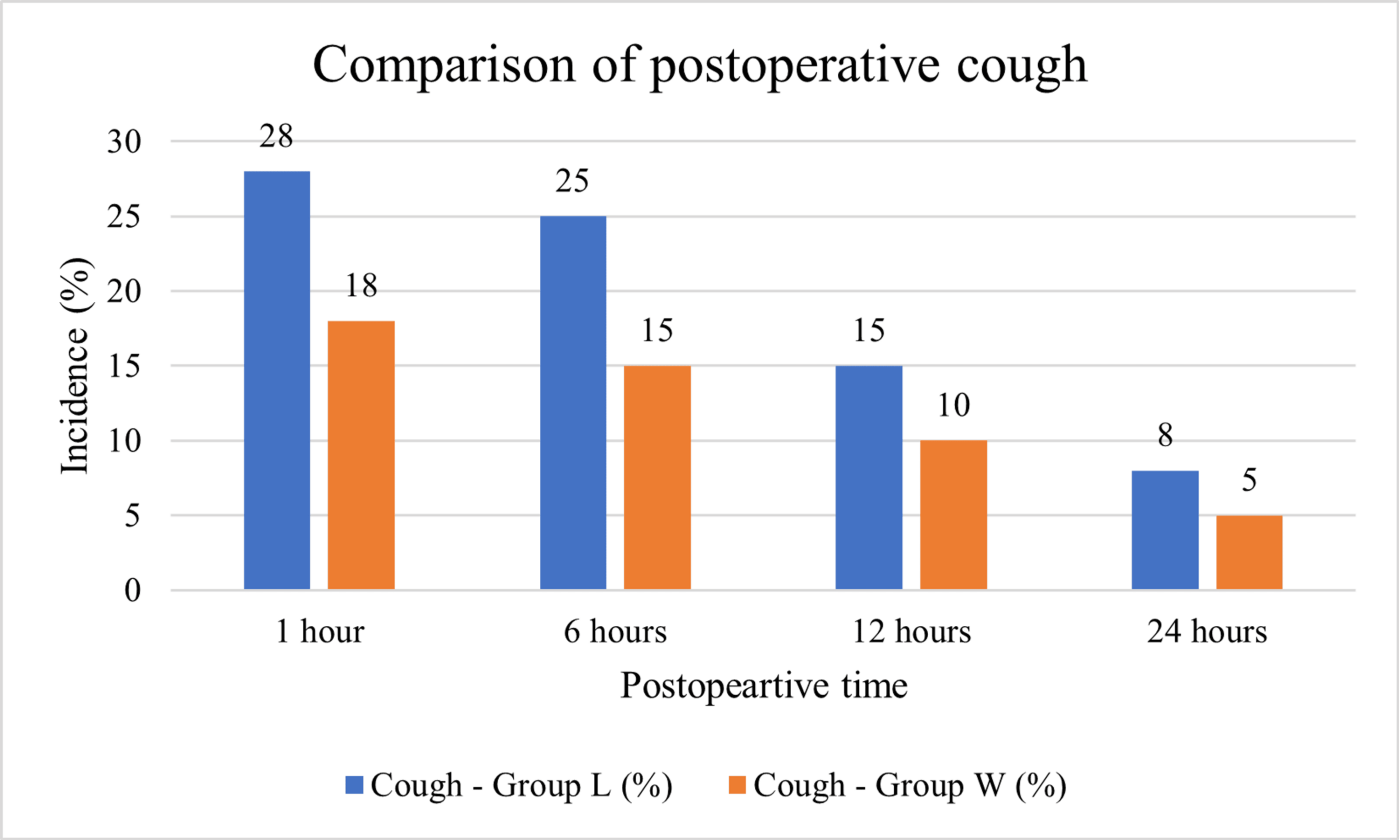
Comparison of the incidence of postoperative cough between lignocaine jelly (Group L) and water-based jelly (Group W) at different time intervals.

Figure 2 compares the incidence of postoperative cough between the two groups (Group L - lignocaine jelly, and Group W - water-based jelly) across different postoperative time intervals (1, 6, 12, and 24 hours). At 1 hour after extubation, the incidence of cough was highest in both groups but markedly more in the lignocaine group (28%) compared to the water-based group (18%). By 6 hours, the incidence of cough reduced in both groups, yet the difference persisted, with Group L showing 25% incidence versus 15% in Group W. At 12 hours, the cough rates further decreased, recorded at 15% in Group L and 10% in Group W, demonstrating a continued advantage of the water-based jelly. By 24 hours, the cough was nearly resolved in both groups, with 8% incidence in Group L and 5% in Group W. The overall trend shows a steady decline in cough symptoms over time in both groups, but consistently higher rates in the lignocaine jelly group at every interval. 

**Figure 3:**
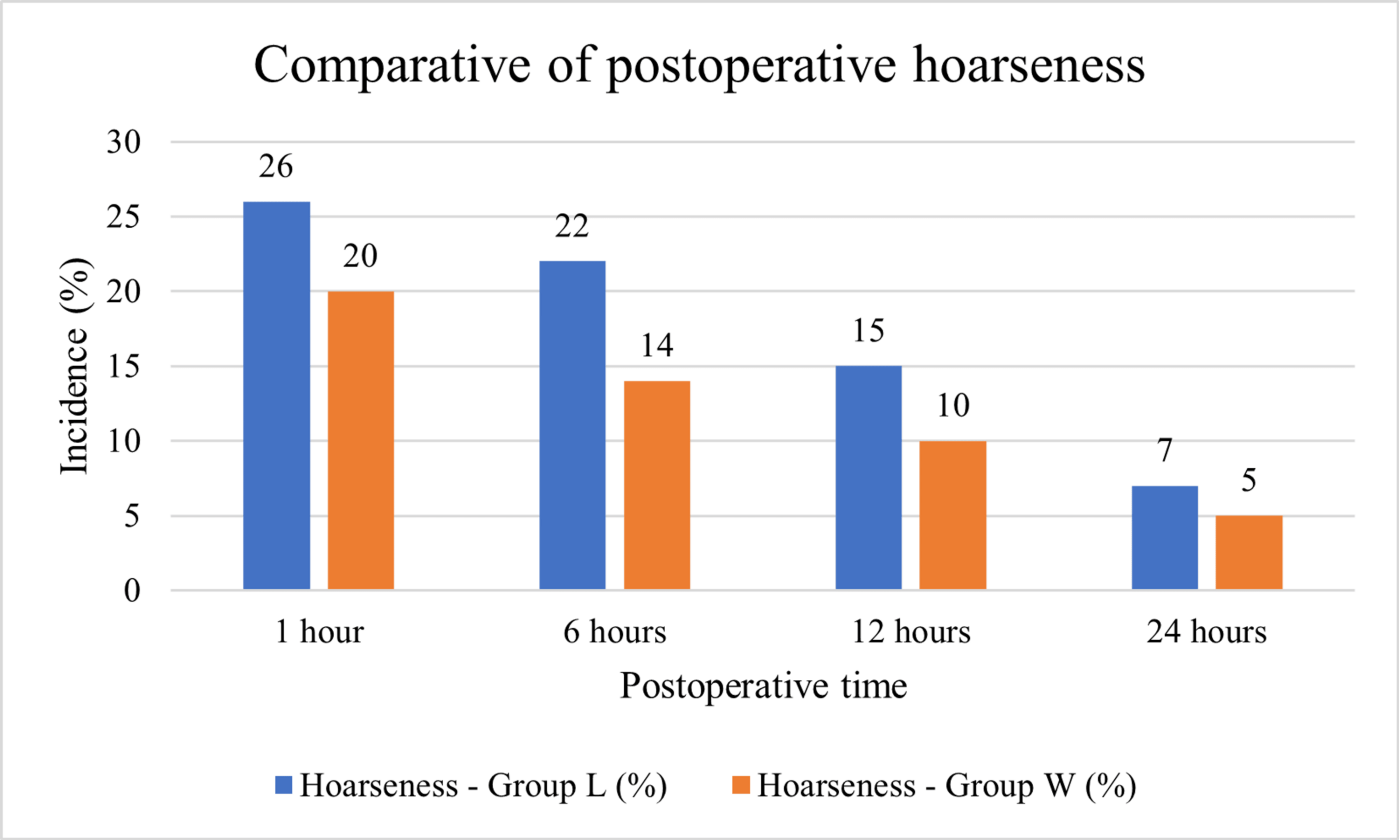
Comparison of the incidence of postoperative hoarseness between lignocaine jelly (Group L) and water-based jelly (Group W) at different time intervals.

Figure 3 illustrates the comparison of postoperative hoarseness between the two groups at different time intervals after extubation. At 1 hour, hoarseness was most frequent in both groups, but its incidence was higher in the lignocaine jelly group (26%) compared to the water-based jelly group (20%). At 6 hours, there was a reduction in both groups, though the difference between them became more apparent. Group L recorded an incidence of 22%, whereas Group W showed 14%. By 12 hours, the symptoms declined further, with 15% of patients in Group L experiencing hoarseness compared to 10% in Group W. At 24 hours, most patients had recovered from hoarseness, with only 7% in Group L and 5% in Group W still reporting symptoms. Although the differences narrowed over time, the overall trend demonstrates that water-based jelly consistently resulted in lower rates of postoperative hoarseness at all measured intervals. 

Across all three outcomes, the lignocaine jelly group consistently showed higher symptom incidence compared to the water-based jelly group, with differences reaching statistical significance in the early postoperative hours and narrowing by 24 hours.

## Discussion

The present prospective comparative study evaluated the efficacy of lignocaine jelly versus water-based jelly in preventing postoperative airway complications such as sore throat, cough, and hoarseness following endotracheal intubation. The two groups were comparable in terms of demographic and baseline characteristics, including age, gender, height, and weight, ensuring that differences in outcomes could be attributed to the intervention rather than patient variability. Our findings demonstrate that postoperative sore throat, cough, and hoarseness were consistently more frequent in the lignocaine jelly group compared to the water-based jelly group at all postoperative intervals. These complications were most pronounced in the immediate postoperative period (1-6 hours) and gradually declined by 24 hours, where both groups showed near resolution of symptoms. However, the incidence remained higher in the lignocaine group throughout, highlighting the relative advantage of water-based jelly. Postoperative sore throat is a well-documented complication of endotracheal intubation, with incidence ranging from 21% to 65% depending on tube size, cuff pressure, lubrication, and duration of intubation [3,15].

In our study, sore throat incidence peaked at 35% in the lignocaine group versus 20% in the water-based group at 1 hour, closely aligning with previous reports. Similar results were observed in studies comparing lubricating agents, where non-medicated gels were shown to reduce mucosal trauma and frictional injury during intubation [7]. The mechanism likely relates to the physical protective coating provided by water-based jelly, which reduces direct mucosal abrasion and mechanical irritation. Cough, another common airway complication, was observed in 28% of patients in the lignocaine group compared to 18% in the water-based group at 1 hour. Earlier studies have suggested that cough may be linked to tracheal mucosal inflammation and sensitivity caused by residual anesthetic agents or mechanical irritation [15]. Although lignocaine possesses local anesthetic properties, its jelly formulation may not provide adequate uniform lubrication, and in some cases, its irritant potential may paradoxically increase mucosal sensitivity [16]. Water-based jelly, on the other hand, acts purely as a lubricant, preventing mechanical trauma without inducing additional irritation, which likely explains the lower incidence observed. Postoperative hoarseness followed a similar trajectory, being highest in the immediate postoperative hours and consistently greater in the lignocaine group (26% vs. 20% at 1 hour). Hoarseness results from vocal cord edema, mucosal trauma, or vocal cord contact during intubation [17]. Our results agree with studies showing that effective lubrication of the ETT reduces friction and pressure-related trauma to the vocal cords, thereby minimizing hoarseness [18].

The clinical significance of our findings lies in demonstrating that simple, non-medicated, water-based lubricating jelly is superior to lignocaine jelly in reducing airway morbidity following intubation. Previous studies have investigated various strategies, including cuff lubrication, topical anesthetics, and anti-inflammatory agents, with mixed results [19,20]. Unlike pharmacological interventions, water-based jelly provides a safe, inexpensive, and universally available option without systemic side effects. A key strength of this study is the well-matched baseline demographics, which minimize confounding effects. Furthermore, serial assessment of symptoms at multiple time intervals allowed us to capture the temporal pattern of resolution. However, limitations include the single-center design, not accounting for the duration of intubation as it was similar in most cases, and the subjective nature of symptom reporting, which may introduce bias. Future multicentric studies with larger sample sizes and objective airway assessments (e.g., laryngoscopic scoring of mucosal injury) would provide further validation.

## Conclusions

This prospective comparative study was conducted to evaluate the efficacy of lignocaine jelly versus water-based jelly in preventing postoperative sore throat, cough, and hoarseness following endotracheal intubation. The two groups were comparable in terms of demographic and baseline characteristics, ensuring that the differences observed could be attributed to the intervention. The findings demonstrated that postoperative airway complications were consistently higher in the lignocaine jelly group compared to the water-based jelly group at all observed intervals. Symptoms were most pronounced in the immediate postoperative period and steadily declined by 24 hours, with near resolution in both groups. However, patients lubricated with water-based jelly experienced significantly fewer incidences of sore throat, cough, and hoarseness, particularly within the first 6-12 hours after extubation. These results highlight that water-based jelly, a simple, inexpensive, and widely available agent, is more effective than lignocaine jelly in reducing airway-related morbidity after intubation. The study thereby fulfills its objective of identifying a practical preventive strategy for common postoperative complaints. Adoption of water-based lubricating jelly as a standard practice can enhance patient comfort, reduce postoperative dissatisfaction, and improve overall quality of anesthetic care.
